# The effect of piperine on midazolam plasma concentration in healthy volunteers, a research on the CYP3A-involving metabolism

**DOI:** 10.1186/2008-2231-22-8

**Published:** 2014-01-07

**Authors:** Mohammad Mahdi Rezaee, Sohrab Kazemi, Mohammad Taghi Kazemi, Saeed Gharooee, Elham Yazdani, Hoda Gharooee, Mohammad Reza Shiran, Ali Akbar Moghadamnia

**Affiliations:** 1 Department of Pharmacology, Babol University of Medical Sciences, Babol, Iran; 2 Department of Pharmacology, Mazandaran University of Medical Sciences, Sari, Iran; 3 Cellular and Molecular Biology Research Centre, Babol University of Medical Sciences, Babol, Iran

**Keywords:** Piperine, Midazolam, CYP3A, Clearance, Half-life, Microsomal hepatic metabolism, HPLC

## Abstract

Some studies showed that piperine (the alkaloid of *piper nigrum*) can change the activities of microsomal enzymes. Midazolam concentration is applied as a probe to determine the CYP3A enzyme activity. This study was done to determine piperine pretreatment role on midazolam plasma concentration.

Twenty healthy volunteers (14 men and 6 women) received oral dose of piperine (15 mg) or placebo for three days as pretreatment and midazolam (10 mg) on fourth day of study and the blood samples were taken at 0.5, 2.5 and 5 h after midazolam administration. The midazolam plasma levels were assayed using HPLC method (C18 analytical column, 75:25 methanol:water as mobile phase, UV detector at 242 nm wavelength and diazepam as internal standard). Data were fit in a “one-compartment PK model” using *P-Pharm 1.5* software and analyzed under statistical tests.

The mean ±SD of the age and body mass index were 24.3 ± 1.83 years (range: 21–28 years) and 23.46± 2.85, respectively. The duration of sedation in piperine receiving group was greater that the placebo group (188±59 *vs.* 102±43 min, p<0.0001). Half-life and clearance of midazolam were higher in piperine pretreatment group compared to placebo [1.88±0.0*3 vs.* 1.71± 0.04 h (p<0.0001) and 33.62 ± 0.4 *vs.* 37.09 ± 1.07 ml/min (p<0.0001), respectively].

According to the results, piperine can significantly increases half-life and decreases clearance of midazolam compared to placebo. It is suggested that piperine can demonstrate those effects by inhibition CYP3A4 enzyme activity in liver microsomal system.

## Introduction

Some specific substances of food products and additives can manipulate drugs disposition and fate in body through mechanisms that make changes their plasma concentration [1-3]. These changes will exert as inducers or inhibitors of hepatic microsomal enzymes or transporters [1,3-5]. Black pepper (piper nigrum) has been used widely throughout the world. It has been shown that pepper can change metabolism of some drugs in those that take foods containing high pepper [6]. Piperine is an isolated alkaloid of black pepper [7] that belongs to capsaicin like family [8]. Animal studies have previously demonstrated that piperine inhibits several enzymatic pathways involving P450, as well as phase II metabolism [9,10].

Grilled meat using wood charcoal, smoked meats, foods containing cruciferous vegetables, and some medicinal plants can induce several microsomal and transporter enzymes and decrease plasma concentration of xenobiotics, such as cyclosporine A and anti-HIV protease inhibitors [11]. Other drugs, such as verapamil induces CYP3A4 and increases transport of p-glycoproteine precursor [11]. On the other hand, some foods such as grape fruit can increase plasma concentration of felodipine, nitrondipine, secoanavir and cyclosporine A by inhibiting of CYP3A [12,13]. It has been suggested that pretreatment piperine in mice, may result in increasing plasma concentration of theophylline [9,14]. This similar effect of piperine on plasma level of theophylline, rifampine, phenytoin and propranolol has been observed in human studies [15]. It has been reported that administration of 1 g of black pepper as a single dose, may increase time-concentration area under the curve (AUC) of phenytoin more than two times compared to placebo.

CYP3A enzymes are the most important enzymatic group that involve in drug metabolism. More than 50% of microsomal enzymes are made up of CYP3A and about 60% of drugs used in treatment (e.g. calcium channel blockers, corticosteroids, sex hormones, macrolide antibiotics, benzodiazepines and immunosuppressant drug cyclosporine) are metabolized by CYP3A [16]. Plasma concentration of some drugs such as midazolam and caffeine can serve as a probe of the enzyme activity [17,18]. Midazolam is a short acting sedative bezodiazepins that is mainly metabolized by CYP3A4 [7]. Determination of plasma concentration of midazolam can help to assess the activity level of CYP3A4 [6].

Based on the mentioned effect of piperine and the important role of CYP3A in drug metabolism, this study was designed to investigate the effect of three days pretreatment of low dose piperine on midazolam plasma concentration in healthy volunteers.

## Materials and methods

This study has been approved by ethics committee of Babol University of Medical Sciences (Babol, Iran) and recorded in IRCT (Iranian registry of clinical trials) data bank with registration number:IRCT201203129271N1. All healthy volunteers were given information of the aims of the study, methods and possible outcomes of the treatment so that they could understand the main objective of the investigation. All selected participants signed a written informative consent form and then they were entered into the study. Although, neither midazolam nor piperine have serious side effects at lower doses, all participants were allowed to leave the study at any time of the follow up when they developed considerable complications.

### Type of study

This was a cross-over controlled study on two groups (n=10) of healthy volunteers. A one-month period was considered as wash out time between two trials. Based on previous reports in 100 healthy volunteers of Mazandaran province (Northern Iran) to find out the CYP3A activity and recorded moderate activity in this sample size of the population [17,18], all participants were selected from Babol, one of the big cities of the province. After providing adequate information to the subjects, eligible healthy volunteers will be enrolled in the study. Inclusion criteria: being healthy; age ranging 18 to 35; normal diet; not receiving microsomal enzyme inhibitor or inducer agents for at least 7 days before the study; not using black pepper for at least 7 days before the study. Exclusion criteria: age being below 18 and above 35; smocking; pregnancy; metabolic disorders; high pepper diet, receiving enzyme inhibitors or inducers.

### Drugs & chemicals

Midazolam (Exir Lorestan, Iran), EDTA (Sigma Chemical Co. USA), piperine (Merck, Germany), n-hexan, HPLC grade methanol, isoamyl alcohol and HCl (Merck, Germany) and deionized water were used. Standard form of midazolam and diazepam as base (used as internal standard) were prepared from Dr. Abidi Pharmaceuitical Company (Tehran, Iran).

### Devices

Centrifuge (Clements 2000, Australia) and HPLC [(Knauer, Germany); column Eurospher 100–5 C18 of silica gel, dimension: 250 × 4.6 mm with pre-column, UV detector, EZchrom Elite software] were used.

### Steps

All participants received adequate description about the setting, treatment schedule and outcome of drug administration. Then they recorded their personal information including age, sex, height, weight, general health situation, and history of drug use, food sensitivity, smoking, occupation, education and consent to the study in a special form.

### Drug administration and sampling

Healthy volunteers regardless of gender were randomly divided into two control and piperine receiving groups (n=20 per group). The subject received placebo or piperine (1 capsule 15 mg of piperine daily for 3 days before midazolam) and midazolam (10 mg as an oral single dose) on fourth day of the study. Total period of the study is 4 days. Blood samples (maximum 10 ml) were taken at 0.5, 2.5 and 5 hour after midazolam administration. After a month as a wash out period, the treatment program was exchanged between placebo and piperine groups and all steps were done as same as the previous.

### Blood samples preparation

Blood samples were transferred into labeled tubes containing Ca-EDTA and shake gently to mix Ca-EDTA with blood for preventing of coagulation.The samples were centrifuged at 3500 ×g for 15 min and supernatant plasma layer transferred into a blank tube. Until the assay of midazolam or diazepam concentration, the plasma samples were kept at −20°C.

### HPLC assay

For measurement of the drugs concentration, plasma samples were removed from the freezer condition and left in the laboratory to convert from frozen into a liquid sample. Then 0.5 ml of thawed plasma sample was transferred into a capped falcon tube. In the next step, 100 micl of 1 M NaOH and 100 micl isoamyl alcohol were added. After a gently shaking, 4.5 ml of n-hexan was added and shake for 1 minute. The final solutions were centrifuged at 300 ×g for 5 min. After this step, 4.2 ml of supernatant was drawn and transferred into a plastic tube and 100 miclof 0.05 M HCl was added to the solution and centrifuged similar as the previous. Then the supernatant (organic layer) was discarded and the remaining was directly injected into the HPLC.

### HPLC analysis

The separation was carried out at ambient temperature using a single-column isocratic reverse-phase method. The mobile phase consisted of 75% methanol and 25% water. Flow rate was set at 0.8 ml/min. The extracts were detected by UV detector at 242 nm wavelength. Twenty micl of the aqueous layer of final extract was injected into the HPLC using a Hamilton’s syringe. The injection was repeated 3 times for each sample. Peaks area and height were considered for midazolam and diazepam detection and used for calculation of the drugs concentration in the final plasma extract.

### Standard preparations

A working solution containing 10 micg of midazolam in 1 mL of mobile phase was prepared. Six standard solutions of different concentrations of midazolam (20, 50, 75, 100, 150 and 200 ng/ml) were prepared. Then 20 micl of 25 ng/ml of diazepam solution as internal standard as added to each standard concentration of midazolam.

### Data handling and analysis

Data were analyzed in two steps. The first; personal information of the participants and data of drugs assay were handled using Excel software. Calibration curves were plotted based on peak area and/or height ratio of midazolam to diazepam.

The slope, intercept and R^2^coefficient were calculated. Coefficient variation (CV) for this analysis was 3.8%.

The second; pharmacokinetic (PK) parameters such as absorption rate constant (Ka), clearance, half-life and volume of distribution of midazolam in the placebo and piperine groups were calculated based on assumed one-compartment kinetics using *P-Pharm* software.

Based on non-parametric distribution, data of three concentrations by time were analyzed using Friedman test. Wilcoxon U-test was used for the results of clearance, volume of distribution and half-life of midazolam in piperine group compared to placebo. The difference of the PK parameters between two groups was considered statistically significant at p<0.05.

## Results

The age range of the participants was from 21 to 28 year (24.3±1.83). Six subjects were female and 14 were male (mean±SD of age: males: 25±1.26, females: 22.66±1.86). All participants were healthy volunteer and selected from Mazandaran province. The subjects’ weight ranged from 49 to 101 kg (68.05±2.85). Their average (±SD) BMI was 23.46 (±2.85).

### Clinical features

All the subjects experienced sedation and mild hypnosis following midazolam administration. This situation was transient and they’ve got improvement during next 4 hours after administration of midazolam. In some subjects, severe sedation and dizziness were observed in placebo and piperine groups (30% and 55%, respectively). The mean duration of the sedation in piperine receiving group was greater than placebo (188±59 *vs*. 102±43 min, p<0.0001) (Table 1).

**Table 1:** Comparison of the mean (±SD) of onset and duration of sedative effect and frequency of sedation and amnesia following midazolam administration in placebo and piperine pretreatment groups (n=20)

	**Onset of drug effect (min)**	**End of drug effect (min)**	**Sedation**	**Amnesia**
Placebo	11.25 (±4.5)	102 (±43)	16	4
Piperine	11.75 (±4.9)	188 (±59)	20	**7**
p value	NS*	0.0001	NS	NS

*NS: non-significant.

**Figure 1:**
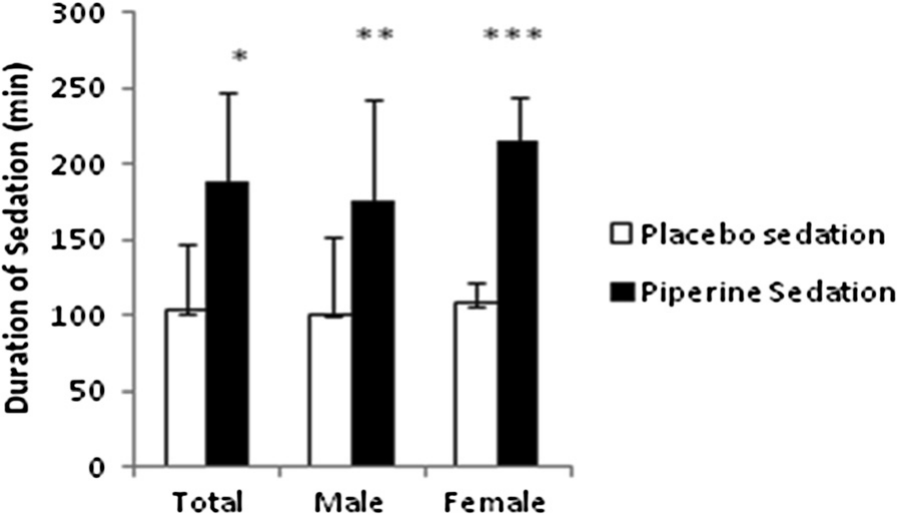
**Comparison of duration of sedation (min) after midazolam (10 mg, p.o.) in placebo and piperine pretreatment (15 mg p.o. in three sequential days before midazolam) groups.** Total data and data by sex are shown for 20 subjects. Sedation in piperine pretreatment group is greater than placebo group (*p<0.001, **p<0.01, ***p<0.0001).

Duration of midazolam sedative effect in piperine pretreatment group in the females was greater than the males when compared to placebo (p=0.027 and p<0.001) (Figure 1). Seven subjects of piperine group showed predominant amnesia but only 4 subjects were amnesic in placebo group. One subject of piperine group showed hiccup when receiving midazolam that was disappeared during first hours after drug administration. The supine position was recommended for participants suffering from severe dizziness and possible hypotension.

### Standard curve

**Figure 2:**
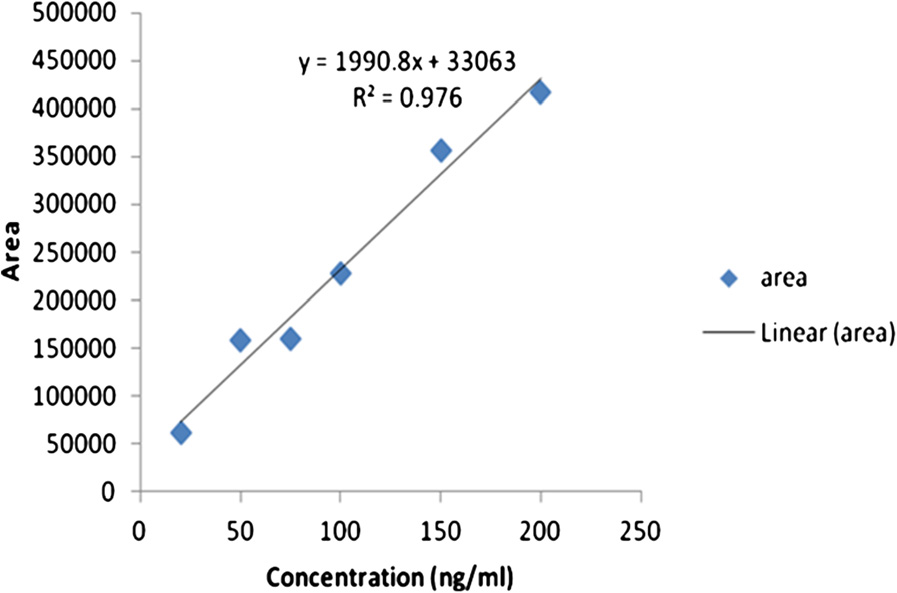
Peak area ratios (midazolam/diazepam)-concentration curve of six different concentrations (ng/ml) of midazolam containing equal diazepam (25 ng/ml) as internal standard.

Six concentrations (20, 50, 75, 100, 150 and 200 ng/ml) as standard solution of midazolam were prepared. Equal volume of diazepam solution (25 ng/ml) as internal standard was spiked on each concentration of midazolam. Based on peak area ratios of different concentration of midazolam on diazepam, the standard curve was obtained (Figure 2). The linearity (R^2^) constant and the line equation are shown in the Figure 2.

**Figure 3:**
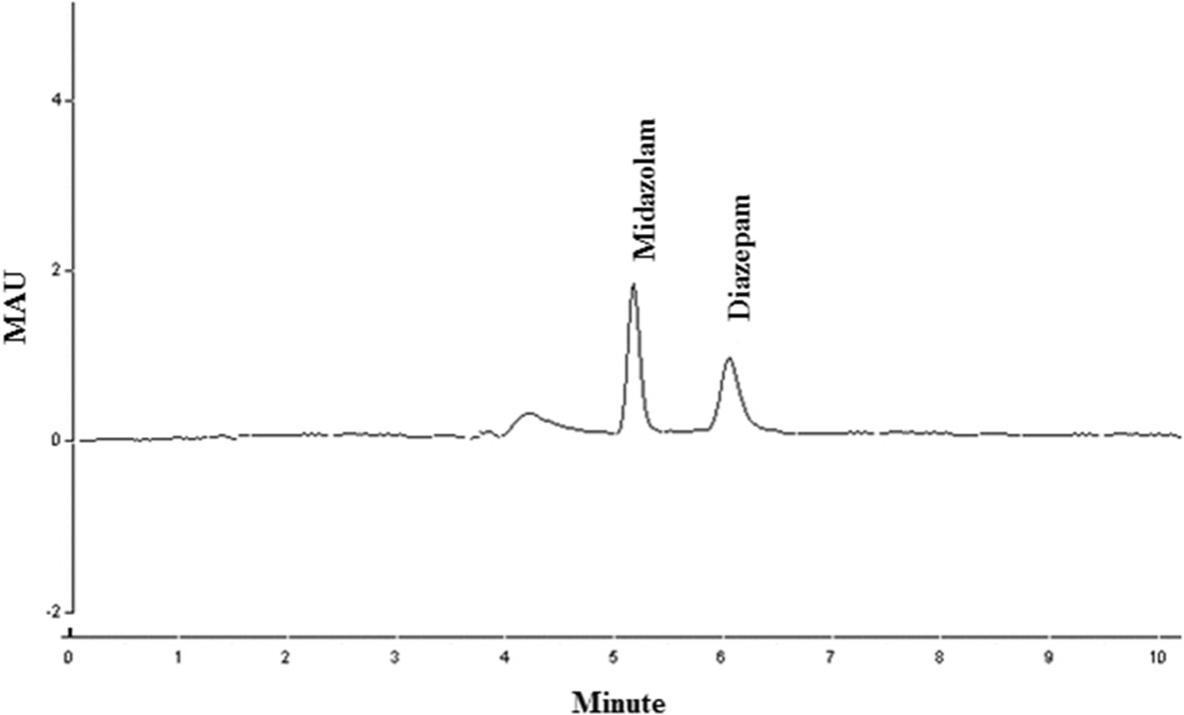
Chromatograms of midazolam standard concentration (50 ng/ml) and diazepam (25 ng/ml).

The chromatograms of standard midazolam (50 ng/ml) and diazepam (25 ng/ml) are shown in Figure 3. Diazepam was used as internal standard.

The comparison of primary data of drug concentrations showed a significant different between placebo and piperine pretreatment groups at 2.5 and 5 hours after midazolam (p=0.015 and p=0.002, respectively). Analysis of intra-group variation of concentration by time showed a significant profile (p=0.03). There was no significant difference in AUC_0-5_ of placebo compared to piperine pretreatment group (411.1± 235.4 *vs.* 495, 9 ± 273, respectively).Pharmacokinetics parameters were estimated in an assumed one-compartment model using *P-Pharm* software. For comparing final estimation, non-parametric paired Wilcoxon U-test was applied.

All PK parameters except of Vd, in piperine pretreated subjects has demonstrated a significant difference compared to placebo (p<000.1). Half-life of midazolam in piperine pretreatment group is considerably greater than in placebo (Table 2). For this reason, it could be said that piperine may decrease metabolism of midazolam.

**Table 2:** Comparison of pharmacokinetics (PK) data of midazolam in placebo and piperine pretreatment groups

**Group**	**Clearance**	**Volume**	**Kel†**	**T1/2‡**
Placebo (n=20)				
*Mean*	37.09	91.44	0.41	1.71*
*Min*	34.27	87.63	0.390	1.63
*Max*	38.48	93.03	0.42	1.78
*S.D.*	1.07	1.54	0.01	0.04
*Fold*	1.12	1.06	1.1	1.1
*C.V.*	2.89	1.69	2.62	2.61
Piperine (n=20)				
*Mean*	33.62	91.43	0.37	1.88
*Min*	32.63	84.45	0.35	1.86
*Max*	34.02	92.35	0.37	1.96
*S.D.*	0.4	1.7	0.01	0.03
*Fold*	1.04	1.09	1.05	1.05
*C.V.*	1.2	1.86	1.29	1.33

†Elimination rate constant.

‡Half- life.

*p<0.0001 (with piperine group).

**Figure 4:**
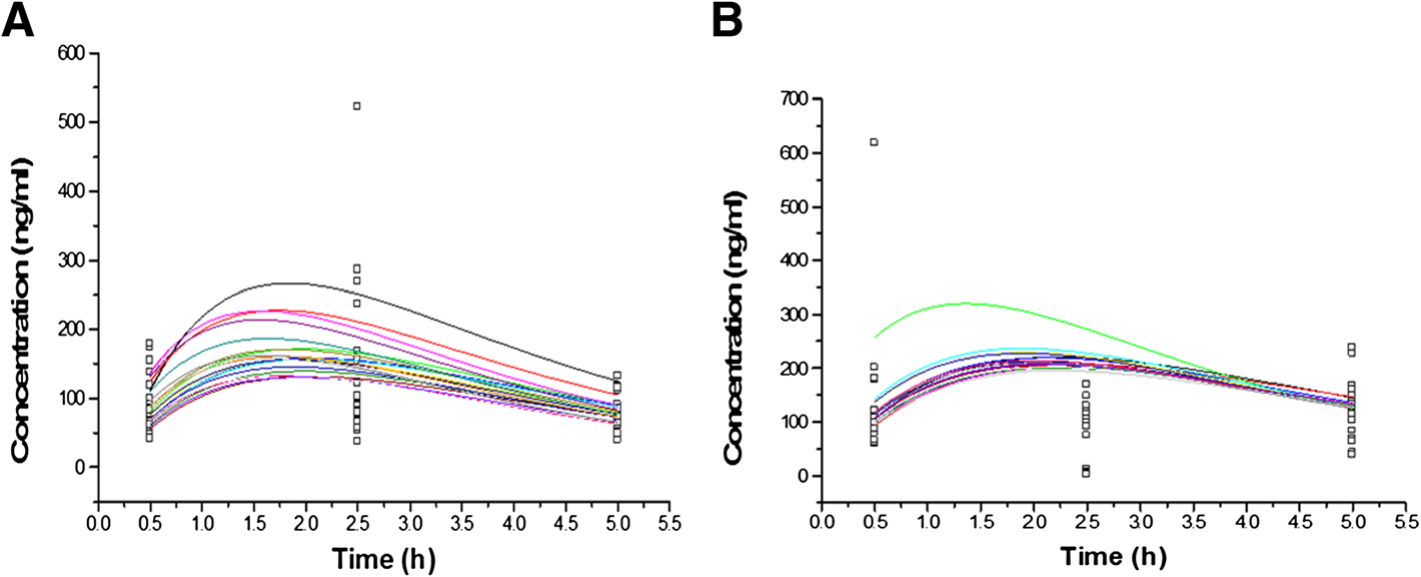
**Bayesian predicted (thin line) and observed (symbols) plasma midazolam concentrations in placebo (A) and piperine (B) receiving groups.** Note the different scales of concentrations in the figures.

After modeling with input of initial data assumption (Cl= 40 ml/min, Vd=80 L, Ka=1, F=1), concentration-time curves were plotted. Individual Bayesian fits of concentration (ng/ml) of midazolam by time (h) profile placebo and piperine pretreatment are shown in Figure 4.

The population plasma midazolam concentrations time curves in placebo (A) and piperine (B) treatment groups are shown in Figure 4. Based on the figure (A), one subject showed an out of range concentration at the time of 2.5 h. It is considered that the maximum concentration in placebo group occurred at 1.5 h after midazolam administration.

## Discussion

This investigation showed 3 days piperine pretreatment can significant increase elimination half-life (t1/2) and decrease clearance of midazolam when compared to control. Our results can demonstrate a possible relation between midazolam concentration and CYP3A activity. This enzyme has an important role in microsomal drug metabolisms. Thirty percent of cytochrome P450 enzymes of liver are made of CY3A4 [19]. It has been estimated that CYP3A4 is responsible for 50 percent of the metabolism of all drugs that being eliminated via hepatic microsomal enzymatic system [20,21]. In addition to having an active role in hepatic metabolism of drugs, CYP3A4 is sufficiently active in the small intestine [22,23]. CYP3A4 is active in the metabolism of lipophilic substrates such as fentanyl, alfentanil, oxycodone, and methadone [24-28]. CYP3A4 inhibitors that have been well studied include: azole antifungal agents and a number of the macrolide antibodies [29]. There are clinically important examples such as midazolam, alprozolam, atorvastatin, simvastatin, felodipine, nifedipine, and cyclosporine that are affected by this system [29]. Among the CYP3A substrates, midazolam has been introduced as a predominant substrate of CYP3A4 and CYP3A5 [29]. The metabolism of intravenous midazolam can reflect to the rate of hepatic CYP3A activity but when it is administered by mouth, the metabolism can demonstrate both liver and enteral origin of CYP3A [22,23]. Any changes in CYP3A enzymatic pathway, may affect midazolam metabolism. CYP3A activity can be affected by different factors such as genetics, nutritional, environmental, and hormonal and disease conditions. These variations in CYP3A activity may create some problems in dosing of the drugs those are metabolized by this enzymatic system. These variations may increase drug interaction episodes and cause side effects to the patients. More than 50% of drugs that are eliminated by liver microsomal enzymes are metabolized by CYP3A system [20,21].

In the present study piperine the main alkaloid of black pepper [7], could significantly prolong life time of midazolam in body. This may increase the pharmacologic activity and can induce and prolong sedative-hypnotic properties of the drug. These effects of piperine may be the result of inhibition of CYP3A4 activity. Previous animal studies have demonstrated that piperine inhibits several CYP450 mediated pathways [30]. Piperine is a selective non-competitive inhibitor of CYP3A but has lower activity on the other microsomal enzymes. It can inhibit activity UDP-glucuronosyl transferase as well [31,32]. Pretreatment of piperine in mice induced higher plasma levels of theophylline, rifampin, phenytoin and propranolol and Q10-CoA compared to control [33,34]. Such an effect has been reported in a previous clinical study [15]. It has been reported that a single dose of 1 g of black pepper can increase the AUC (area under the curve of plasma concentration-time) of phenytoin [14]. In the present study piperine was given in low dose and seems that in this dose, it can change important PK parameters such as clearance and t/12 of midazolam. It means that piperine’s direct effect on midazolam elimination is not assumed and it can increase the half-life of midazolam via inhibition of its hepatic microsomal elimination. This may increase duration and severity of side effects of subject drugs (substrate of CYP3A) following their elevated plasma levels. As we already showed the midazolam side effects in piperine pretreated subjects were considerable compared to placebo. The inhibition or induction of enteral CYP3A4 and p-glycoprotein can mediate considerable drug interactions. These of interactions may be due to a considerable variation in drug action from no effect to toxic effect of drugs [35-38].

The genes of CYP3A4 and p-glycoprotein are expressed in enterocytes and the bioavailability of many drugs such as cyclosporine A, midazolam, verapamil, HIV protease inhibitors and digoxin could be affected by the enzyme and transporter [11,36,38-40]. Metabolism of midazolam when prescribed intravenously shows the activity of hepatic CYP3A, but when it is prescribed orally, it can show the activity of both hepatic and intestinal CYP3A [41,42]. After oxidative metabolism, midazolam changes into one of its main metabolites, hydroxide midazolam, in liver, and is mediated in an exceptional rout by CYP3A isoforms [43,44]. Piperine inhibits p-glycoprotein and CYP3A activity. Since, these proteins become expressed in enterocytes and hepatocytes; they have an effective role in first-pass metabolism of subject drugs. Piperine content of nutritional regimen can change the levels of p-glycoprotein and CYP3A substrates in blood when those administered by mouth [45]. However more researches are needed to prove intestinal or hepatic effects of piperine, especially to justify their mechanism in the present study. In this investigation duration of sedation and possible hypnosis action of midazolam in females was longer than the males. This may be to the result from higher plasma level of midazolam after piperine pretreatment in female subjects. Perhaps, it is concluded that sex may be an important factor to obtaining plasma concentration of midazolam. This may be due to higher activity of CYP3A4 in female at normal condition compared to male [46-49]. In a previous phenotyping study by the authors, similar results by sex variability on CYP3A activity has been reported [17]. For this reason more clinical effects are expected in females if the enzyme becomes inhibited.

## Conclusion

Individual PK variations can significantly determine CYP3A activity in general population. This may predispose patients to demonstrate unexpected drug manifestations compared to normal subjects. According to the present investigation, piperine may inhibit CYP3A4 activity and increase the level of midazolam as a substrate of the enzyme.

## Competing interests

The authors declared that they have no competing interests.

## Authors’ contributions

MMR: data collection and executive affairs; SK: laboratory works and review literature searching; MTK: lab tests and data collection; SGh: HPLC model setting; EY and HGh: data collection; MRS: study design and modeling; AAM: study design, to register study in IRCT, data handling and statistical analysis, manuscript writing and correction. All authors read and approved the final manuscript.
